# Intermediate Grade Salivary Gland Mucoepidermoid Carcinoma: Is Neck Dissection Indicated?

**DOI:** 10.1002/ohn.1202

**Published:** 2025-04-04

**Authors:** Jake Langlie, Nicholas DiStefano, Carmen Gomez‐Fernandez, Jaylou Velez‐Torres, Jason Leibowitz, David Arnold, Donald Weed, Francisco J. Civantos

**Affiliations:** ^1^ Department of Otolaryngology, Division of Head and Neck Surgery University of Miami Miller School of Medicine Miami Florida USA; ^2^ University of Miami Miller School of Medicine Miami Florida USA; ^3^ Department of Pathology, Division of Head and Neck Pathology University of Miami Miller School of Medicine Miami Florida USA

**Keywords:** intermediate grade, malignancy of salivary origin, metastasis, mucoepidermoid carcinoma, neck dissection, parotid cancer

## Abstract

**Objective:**

NCCN guidelines recommend a neck dissection addressing at least levels II‐IV for high‐grade mucoepidermoid carcinoma (MEC) and close observation of the lymphatic basins for low‐grade MEC. However, no guidelines exist for intermediate‐grade MEC with clinically and radiologically uninvolved cervical lymph nodes.

**Study Design:**

Retrospective analysis.

**Setting:**

Patients with intermediate‐grade MEC with a clinically N0 neck from our tertiary academic institution from 2015 to 2023.

**Methods:**

Evaluation for histologic lymphatic metastases was performed when surgeons elected to perform neck dissection. For patients who did not receive a neck dissection, review of medical records to document the results of clinical observation, and specifically regional lymphatic recurrence, on long‐term follow‐up.

**Results:**

Thirty‐five patients with N0 intermediate grade MEC were included, composed of 26 patients who underwent primary tumor resection and neck dissection and 9 patients who received resection of the primary tumor without neck dissection. One out of 26 patients receiving neck dissection was found to have lymphatic metastasis. Watchful waiting of 9 patients demonstrated no recurrence at a mean follow up of 40 months. Thus, 1 out of 35 patients (2.9% [95% confidence interval: 2.7%‐3.1%]) had documented metastatic disease in the lymphatics.

**Conclusions:**

For patients presenting with intermediate‐grade MEC, there was a low chance (2.9%) of positive histologic or clinical lymphatic metastases in the neck. Given this low risk, we believe the potential benefit of neck dissection may be outweighed by the potential morbidity. Careful consideration of the clinical behavior of the lesion could be considered along with a more selective approach toward elective lymphadenectomy in intermediate‐grade MEC.

Salivary gland tumors encompass a wide variety of neoplasms categorized into benign and malignant subtypes.^1^ Treatment of the neoplasm varies significantly based on type, size, location, stage, and presence of metastases. Mucoepidermoid carcinoma (MEC) is the most common malignant tumor of the major salivary glands. It primarily develops in the parotid and the submandibular glands, with decreasing incidence within the minor salivary glands from the mouth to the subglottis.^2^ Risk factors for MEC include prior radiation exposure, smoking, and consumption of processed meats.^3^, ^4^ Treatment of MEC can vary from wide excision of the primary tumor alone to wide excision with lymphadenectomy and/or radiation therapy.

Adults with low‐grade MEC have a 5‐year survival rate of approximately 98.8%.^5^ These tumors are more commonly cystic or well‐encapsulated and can often be cured with local surgical excision. Low‐grade MECs are less aggressive and often have an excellent prognosis. Treatment typically involves local surgical excision with a small margin of healthy tissue to ensure complete removal of the tumor. This approach minimizes recovery time and reduces the risk of complications. Radiation therapy is generally not required unless there is a concern about incomplete excision or close margins. Close observation of the lymphatic basins is recommended, but formal neck dissection is usually not necessary.

Adults with high‐grade MEC have a 5‐year survival rate of around 67.0%.^5^ High‐grade MECs are aggressive and require extensive treatment. This typically includes wide surgical excision to ensure clear margins, elective neck dissection, and postoperative radiation therapy to control local disease and reduce recurrence risk. In cases where there is clinical or radiological evidence of lymph node involvement, a neck dissection is always performed. Elective neck dissection (END) is generally also indicated for N0 patients without clinical evidence of lymph node metastases due to the associated 30%–50% risk of occult metastases in high‐grade MEC.^6^ The National Comprehensive Cancer Network (NCCN) guidelines recommend addressing at least levels II, III, and IV during neck dissection for high‐grade tumors.^7^, ^8^ Postoperative radiation is usually needed. Additionally, chemotherapy may be considered in cases where the tumor is particularly aggressive or has spread beyond the primary site.

Adults with intermediate‐grade MEC have a 5‐year survival rate of about 97.4%.^5^ Management strategies for intermediate‐grade tumors vary, highlighting a significant gap in standardized treatment protocols with no clear consensus on management strategies. A neck dissection may be performed when there is clinical evidence of regional metastasis, high TNM stage, or mixed histopathological features.^9^ These tumors have a prognosis and biological behavior that falls between low‐grade and high‐grade tumors.^10^ In the absence of clear guidelines, some clinicians might advocate for a more aggressive approach, including END and postoperative radiation, especially if other high‐risk factors are present. Others recommend a more conservative approach with close monitoring, particularly if high risk clinical or pathological features are not identified.^10^ This lack of clear guidelines highlights the need for further research to establish standardized treatment protocols for intermediate‐grade tumors. The management of the neck for intermediate‐grade MECs remains controversial, but a pragmatic approach might be to consider a neck dissection if there are other high‐risk factors or if the patient is requiring violation of the neck for reconstruction including free flap vessel exposure.

The treatment of MEC, as the most common malignant salivary gland tumor, exemplifies the need for careful grading and treatment planning to optimize outcomes.^11^ This study aims to examine the rate of occult malignancy in clinically and radiologically N0 necks in cases of intermediate grade mucoepidermoid carcinoma at a single, tertiary referral center. Our goal in this study is to evaluate the risk of harboring occult metastases within the cervical lymphatics in patients with intermediate grade mucoepidermoid carcinoma of salivary origin.

## Methods

This retrospective study was conducted from our tertiary academic institution and approved by the Institutional Review Board and Ethics Committee #20110664. Patient records from 2015 to 2023 were reviewed to identify cases of intermediate grade mucoepidermoid carcinoma of the salivary glands with a clinically and radiographically N0 neck. At our institution, mucoepidermoid carcinoma tumors were graded low, intermediate, or high grade based on the most recent WHO guidelines (5th edition) for the management of salivary gland malignancy.^12^ Intermediate grade mucoepidermoid carcinoma is characterized by a nearly solid composition rather than cystic, intermediate cells predominating over mucous cells and mild to moderate cytologic atypia.^10^ The diagnosis of intermediate grade mucoepidermoid carcinoma on final pathology was confirmed by fellowship trained or dedicated head and neck pathologists at our institution on secondary review. In contrast, the presence of mitosis, necrosis, lymphovascular invasion or perineural invasion shifted the diagnosis to high grade mucoepidermoid carcinoma.^12^

Patients with intermediate grade mucoepidermoid carcinoma were identified using Slicer Dicer within the Epic Software^(T)^. Information gathered included patient demographics (age, gender, and prior radiation therapy), tumor characteristics (size, location, TNM stage, and histological grade), surgical details (type of neck dissection, number of nodes dissected, and number of positive nodes), and follow‐up data (recurrence rates, survival rates, and adjuvant treatments). The number of positive lymph nodes was recorded for each patient, and node positivity was calculated based on the ratio of positive nodes to the total number dissected. Statistical analysis was performed using SPSS.

## Results

### Inclusion and Refinement of Patients

A total of 40 patients were initially reviewed for inclusion in the study. Three (n = 3) patients were excluded due to having mixed high‐grade and low‐grade features. Two patients (n = 2) were excluded that had clear radiographically positive lymph nodes in the cervical lymph chain on computerized tomography with contrast and had cervical lymphadenectomy based on postive imaging. A total of 35 patients from 2015‐2023 presenting with intermediate grade, salivary mucoepidermoid carcinoma with clinical stage N0 necks (no evidence of cervical metastases on physical examination or imaging), were studied. To our knowledge, this is the first study that has focused on the cervical metastatic rate in only intermediate grade mucoepidermoid cancers.

These 35 patients were composed of 9 patients who did not undergo a neck dissection, 20 patients with a primary resection and neck dissection, 3 patients with secondary surgeries at our institution where a neck dissection was performed later after the primary surgery, and 3 patients who had their primary surgery or biopsy outside of UM, with full pathology report available for review, and had their follow up completion primary tumor resection and concurrent neck dissection performed at our institution.

### Patient Demographics and Surgical Features

There was a total of 24 females (69%) and 11 males (31%) included in the study with the age of participants varying from age 18 to 84 with a mean of 52.3 ± SD: 15.6 years. Participants were demographically categorized as white, Hispanic (n = 15, 42%), followed by white, non‐Hispanic (n = 9, 26%), black, non‐Hispanic (n = 9, 26%) and Asian, non‐Hispanic (n = 2, 6%). Patient selection for neck dissection versus neck observation was based purely on surgeon decision and preference, and the two groups are not matched demographically. As seen in Table 1, there was a higher percentage of black patients in the neck dissection group, and a lower percentage of white patients. The mean age in the observation group was 52.4 ± 14.9 years and in the neck dissection group was 52.2 ± 16.4 years.

**Table 1:** Demographics and Patient Characteristics: Gender, Age, Race, and Tumor Grade stratified by Neck Dissection Status

Gender (n = 35)	Resection only (n = 9)	Resection + SLND (n = 26)	Total (n = 35)
Male	3 (33%)	8 (31%)	11 (31%)
Female	6 (67%)	18 (69%)	24 (69%)

Age (n = 35)	Resection Only (n = 9)	Resection + SLND (n = 26)	Total (n = 35)
10‐19	.	1 (4%)	1 (3%)
20‐29	.	2 (8%)	2 (6%)
30‐39	2 (22%)	3 (12%)	5 (15%)
40‐49	2 (22%)	6 (23%)	8 (24%)
50‐59	1 (11%)	3 (12%)	4 (12%)
60‐69	3 (34%)	7 (27%)	10 (29%)
70‐79	1 (11%)	3 (12%)	4 (12%)
80‐89	.	1 (4%)	1 (3%)
Mean age +/‐ standard deviation	52.4 + /− 14.9 years	52.2 + /− 16.4 years	52.3 + /− 15.8 years

Race (n = 35)	Resection Only (n = 9)	Resection + SLND (n = 26)	Total (n = 35)
White (Hispanic)	5 (56%)	10 (39%)	15 (42%)
White (non‐Hispanic)	3 (33%)	6 (22%)	9 (26%)
Black (non‐Hispanic)	1 (11%)	8 (31%)	9 (26%)
Asian (non‐Hispanic)	.	2 (8%)	2 (6%)

Tumor Grade (n = 35)	Resection Only (n = 9)	Resection + SLND (n = 26)	Total (n = 35)
T1	6 (67%)	11 (42%)	17 (49%)
T2	1 (11%)	6 (22%)	7 (20%)
T3	1 (11%)	7 (27%)	8 (22%)
T4a	1 (11%)	2 (8%)	3 (9%)

### Tumor Staging

We stratified the surgeries based on T stage and the decision to perform a neck dissection (Table 1). Most tumors in both groups were T1.

### Tumor Location and Neck Dissection Levels

Only patients with a clinically N0 neck at presentation were included, and the primary tumor locations included the parotid gland (26 patients), submandibular gland (2 patients), and minor salivary glands (7 patients composed of 4 base of tongue and 3 maxilla/palate). The most common neck dissections performed included level 1 to 4 (n = 10), including 7 parotid tumors, one submandibular tumor, and 2 base of tongue tumors.

### Lymph Node Metastases

In all, 856 nodes were evaluated using histopathology for the presence of metastatic disease. Each patient had on average 27 nodes assessed (range 5‐96, SD: 21). In total, 1 out of 26 patients undergoing neck dissection (4.0%) and 1 out of 35 total patients in the study (2.9% (95% confidence interval: 2.7%‐3.1%) were found to have evidence of lymphatic metastasis on neck dissection (n = 1) or clinical follow‐up (n = 0). This result is based on one patient who on surgical pathology had one positive node of microscopic disease <0.1 cm with extracapsular extension in the case of a parotid intermediate grade MEC (80% cystic and 20% solid consistency). No other patient with intermediate grade mucoepidermoid carcinoma had a cervical lymph node metastasis in the surgical pathology of the neck dissection in our series, nor did any patient develop a metastasis in the cervical lymphatics on clinical follow‐up.

### Intraparotid Nodes

Regarding intraparotid nodes, 23 out of 26 patients had identified intraparotid lymph nodes (88%) on parotidectomy. 131 intraparotid lymph nodes were evaluated using histopathology. Positive lymph nodes were identified in 2 cases of patients that had not undergone a neck dissection (2/5 patients with identified intraparotid lymph nodes). The first of these had a 1.2 cm intermediate grade mucoepidermoid carcinoma with close margins at the ear cartilage (1 mm), with no other high risk features. Two adjacent micrometastases were encountered in parotid nodes. This patient had indications for radiation based on the close margin and was treated with radiation to the parotid region and external auricle as well as complete radiation field to the cervical lymphatics. Since the neck received radiation, neck dissection of levels 1 through 4 was not performed. The second patient had a 1.7 cm intermediate grade mucoepidermoid carcinoma, with angiolymphatic invasion identified in the primary tumor and closest margin was 1 mm. One micrometastasis was found in an adjacent intraparotid node. Again there were multiple indications for postoperative irradiation, and the lymphatics of the neck (levels 1 through 5) were included. No cases of patients undergoing neck dissection with parotid primary tumors had positive intraparotid lymph nodes, a finding of undetermined significance and probably coincidental. If we include the two patients with intraparotid metastases which were removed during the primary site parotidectomy, 3 out of 35 patients had lymphatic micrometastases (8.5%). However, it should be noted that neither patient required completion neck dissection, and both met criteria for radiation based on the characteristics of the primary site tumor.

### Postoperative Recurrence

Close follow‐up was performed for patients who did not undergo a neck dissection (n = 9), including five tumors located in the parotid, three in the maxilla/palate, and one in the oral tongue. All nine patients at the time of maximum follow‐up per chart review did not have radiographic or clinical evidence of recurrence (6‐106 months with a mean of 40 months and SD of 35.3 months). Three patients did not receive adjuvant radiation. Two out of nine patients underwent radiation to the primary field at a dose of 60 to 66 Gray, without irradiation of the lymphatic basins. Four of nine, received radiation to the neck. Presumably, microscopic disease in the neck could have been controlled by radiation in these cases, but the decision for radiation was based on the characteristics of the primary tumor. All patients included had negative surgical margins at the time of primary resection and all patients remained N0 on postoperative imaging at the time of planning radiation and subsequent to the radiation.

### Review of Outside Pathology and Cytology

Outside histopathology was available and reviewed for 15/35 cases, and outside cytology based on fine needle aspiration was available for 2/15 cases. Mucoepidermoid carcinoma, intermediate grade, was the diagnosis determined in 8/17 cases on review of outside histopathology. Remaining diagnosis given on outside histopathology include: mucoepidermoid carcinoma, low grade (n = 4), mucoepidermoid carcinoma (ungraded) (n = 1), low to intermediate grade carcinoma (n = 1), and squamous mucosa (n = 1). Outside review of cytology determined a diagnosis of suspicious for malignancy (n = 1) and negative for malignancy and suggestive of benign neoplasm (n = 1).

Three patients had an initial surgery at an outside institution that failed to clear the margins and repeat resection at UM produced no additional tumor or microscopic disease. All 14 cases undergoing primary resection at our hospital without prior surgery resulted in a final diagnosis of mucoepidermoid carcinoma, intermediate grade from our tertiary institution's histopathology. The same diagnosis was made on the three patients undergoing repeat resection based on review of outside histopathology. A synopsis of outside pathology review is provided in Table 2.

**Table 2:** Review of Outside Pathology, FNA Status, and Frozen Section Diagnosis

Outside pathology diagnosis	Case number (n = 17)
Mucoepidermoid Carcinoma, Intermediate Grade	8
Mucoepidermoid Carcinoma, Low Grade	4
Mucoepidermoid Carcinoma (ungraded)	1
Outside FNA**—**suspicious for malignancy	1
Low to intermediate grade carcinoma	1
Negative for malignancy, suggestive of benign neoplasm	1
Squamous Mucosa	1

FNA data: Milan grade	Case Number (n = 35)
I	2
II	1
III	1
IVa	1
IVb	1
V	3
VI	5
FNA not performed	17
Biopsy performed (incisional n = 3, core n = 1)	4

Frozen section	Case number (n = 35)
Mucoepidermoid diagnosis, low grade	2
Mucoepidermoid diagnosis, intermediate grade	5
Mucoepidermoid diagnosis, high grade	1
Mucoepidermoid diagnosis, grade not given	7
Frozen only used for margin status	11
Frozen not performed	9

### FNA Results

For cases that did not have review of outside pathology, FNA or core biopsy was often performed. 12/18 cases that had workup performed at our tertiary academic institution underwent FNA and 2/17 cases with outside pathology underwent FNA. The Milan grading system was used to determine likelihood of malignancy and yielded the following grading results: 1 (n = 2), 2 (n = 1), 3 (n = 1), IVa (n = 1), IVb (n = 1), V (n = 3), and VI (n = 5). Four of eighteen cases underwent tissue biopsy (three were incisional biopsy and one was a core biopsy). Two cases did not undergo preoperative biopsy or FNA with one patient declining recommended FNA and the other electing to proceed directly to surgical resection with surgeon agreement. FNA results are highlighted in Table 2.

### Frozen Section Results

For cases where frozen sections were sent for a pathologic diagnosis (n = 15), all cases yielded a diagnosis of mucoepidermoid carcinoma at the time of frozen section. Five of fifteen of these cases that were sent a pathologic diagnosis were reported as intermediate grade to the surgeon intraoperatively while 7/15 cases were reported as mucoepidermoid carcinoma without a grade provided. At the time of frozen, 2/15 cases were reported as low grade mucoepidermoid carcinoma while 1/15 cases were reported as high grade mucoepidermoid carcinoma. For the remaining 20 cases included in the study, 11 cases had frozen sections utilized only for margin status and 9 cases did not have frozen section performed (Table 2).

### Tumor Size

Out of the 35 cases presented in this study, 3 patients had involved lymph nodes with 2 patients with parotid primary tumors having adjacent positive intraparotid nodes and 1 patient having a positive regional neck node. The two patients with positive intraparotid nodes had a tumor with largest dimension of 1.2 and 1.7 cm. The patient with a regional metastasis to the neck had the largest primary tumor in our study at 5.0 cm.

## Discussion

In our study, for patients presenting with intermediate grade mucoepidermoid carcinoma of salivary origin, there was a low chance (4% of dissected necks and 2.9% of the total group) of positive histologic cervical metastases or clinical metastases in the cervical lymphatic basin below the parotid. If patients who received no elective neck dissection but did receive radiation to the neck, are excluded, this leaves us with 1/31, or 3.2% rate of lymphatic metastases. It is reasonable to assume that the two patients who had micrometastases in the parotid, and subsequent radiation, might have had micrometastases to the more inferior cervical lymphatics. Including these patients as positives would give us 3/35 or an 8.5% metastatic rate. Given this relatively low risk, we believe the potential benefit of neck dissection in patients with intermediate grade mucoepidermoid carcinoma may be outweighed by the potential morbidity in selected patients, particularly if the tumor is of early T stage and clinical features do not suggest aggressive behavior of the tumor. However, the small size does not permit the power to run statistical analysis or to draw conclusions for intralesional positive nodes.

Since cervical metastases may share the indolent behavior and intermediate grade pathology of the primary site, “watchful waiting” may be a considered a reasonable alternative in these patients. The information regarding tumor grade may be imperfect at the time of surgery,^13^ as the most accurate determination is on final pathology. However, surgeons must try to distinguish high, intermediate, or low grade as best they can in real time, in order to make a decision regarding lymphadenectomy. This is important since, even though the guidelines are unclear for intermediate grade tumors, there are clear differences in the guidelines between high‐ and low‐grade tumors. The surgeon must put together the limited information from clinical history, intraoperative and preoperative physical findings, imaging, cytology, and frozen section results, and make the best determination they can as to the likely grade of the tumor and indications for formal lymphadenectomy^13^

All 9 patients that did not have a neck dissection did not have recurrence at 42, 6, 7, 13, 66, 36, 12, 106, and 74 months, respectively, that were followed at our institution. Based on our results, we conclude that patients who do not need the neck explored for another reason, including free flap reconstruction, could potentially be considered for an observational approach with respect to the cervical lymphatics. Careful consideration of clinical behavior of the primary lesion and detailed review of pathology could be performed, along with a more selective approach toward formal lymphadenectomy. In the infrequent cases with parotid primary tumors where microscopic lymph node involvement is encountered in the parotid itself, completion neck dissection at a second sitting versus radiation could be offered, depending on whether there are other indications for radiation. In a recent systematic review of salivary gland malignancy evaluating 29 studies composed of 2767 patients, patients with high grade malignancy demonstrated a greater risk of metastasis to local neck nodes of 25.4%.^3^ We could not tease from this systematic review a rate of cervical metastases for intermediate grade tumors. To our knowledge, our study represents the first study critically evaluating the incidence of lymphatic metastases in patients with intermediate grade MEC. It is important to note that this is an observational study of a single referral center and ideally our findings would be confirmed by further studies with larger sample sizes to determine the true risk and benefit of undergoing or not undergoing a neck dissection in intermediate grade MEC.

If we look at what has happened with the management of the cervical lymphatics in a different tumor type, for comparison, there have been significant efforts in oral cavity cancer to reduce morbidity and still stage the lymphatics through the use of sentinel node biopsy, a procedure involving perilesional injection of a radiotracer, and more selective lymph node sampling based on gamma radioactivity.^14^ The sentinel node technology has not been developed for salivary gland tumors, where perilesional injection is generally more difficult. Nonetheless, the existence of this literature for oral cavity cancer points to the importance of reducing neck morbidity by selecting patients who are unlikely to harbor lymphatic metastases. Generally, in oral cavity cancer, and other tumor types, a metastatic rate of less than 10% was considered low enough to justify surveillance instead of formal lymphadenectomy.^15^ For our patients with intermediate grade mucoepidermoid carcinoma, the metastatic rate is below this cutoff. Other studies have recently evaluated the need for elective neck dissection for selected patients with low T stage oral cavity cancers, highlighting the importance of critically evaluating the pathological characteristics and depth of invasion of tumors.^16^ Extrapolating from these efforts in oral cavity cancer, a more common disease, it is important to revisit our approach to the cervical lymphatics for intermediate grade mucoepidermoid carcinoma and consider the benefit of “watchful waiting” in selected patients.

In general, an important consideration in the decision regarding whether to perform elective neck dissection, is the difficulty of re‐exploration if the patient were to develop lymphatic metastases. In the case of parotid tumors, as an example, a very complete parotidectomy**—**which serves as a local lymphadenectomy for intraparotid lymph nodes ‐ is generally recommended for malignancy. We agree that this procedure should be performed in all cases, as re‐exploration in the region of the facial nerve would be very difficult. However, if the neck below the parotid is not disturbed, it is generally not considered difficult to perform a neck dissection secondarily, using the digastric muscle as an upper landmark, and it is these lower cervical nodes that are in question in our study. Given that delayed neck dissection is not technically difficult and based on our data with low rates of cervical lymph node involvement, the surgeon, could consider observing the cervical lymphatics for selected patients with intermediate grade mucoepidermoid carcinoma.

When reviewing outside pathology sent to our institution, the greatest limitation on accurate diagnosis for review of outside pathology was the large variance in slides provided as well as the quality of the slides (slide number provided ranged from 1‐17). Therefore, diagnosis of mucoepidermoid carcinoma was only achieved preoperatively in 13/17 cases with 8/17 cases giving a diagnosis of intermediate grade mucoepidermoid carcinoma.

FNA can help rule in a diagnosis of malignancy but can make it difficult to accurately rule out a diagnosis of malignancy due to the small sample of possible tumor that is blindly collected via this methodology. Among our 12 cases that underwent FNA prior to primary resection at our institution and 2 patients who had repeat FNA after outside pathology review, the majority were either diagnosed as likely for malignancy (Milan Grade V, n = 3) or malignancy (Milan Grade VI, n = 5). However, it is important to highlight the high false negative rate that is observed among FNA biopsies, including 6 patients who were found to have Milan Grade I‐IVa on biopsy. Through the study, we found that 4 patients that underwent either core (n = 1) or incisional (n = 3) biopsy all had a diagnosis of mucoepidermoid carcinoma. Although these are more invasive procedures, and traditionally have raised concerns of soft tissue seeding by tumor, it is important to note that accurate diagnosis was achieved in all cases that underwent tissue biopsy for preoperative workup versus 8/14 cases that were diagnosed as likely for malignancy or malignant on FNA.

In terms of frozen section analysis, all cases that were sent for pathologic diagnosis yielded a diagnosis of mucoepidermoid carcinoma on frozen section. However, only 5/15 of the samples were reported as intermediate grade on frozen section with the majority having the grading deferred to permanent section (7/15 samples). It is well known that it is difficult to assess the grading of mucoepidermoid carcinoma on frozen section with final pathology often aiding in the determination of grade of the lesion.

The retrospective design of this study implies inherent limitations. Results presented in the study were based on the lower quality of data that can be obtained from a patient chart, and there is risk of inaccurate documentation. This was controlled by retrieving data from multiple aspects of the patient chart including clinical visits, pathology reports, and operative reports. As this was a retrospective observational study, the true risk of not undergoing a neck dissection for patients with intermediate grade MEC cannot be determined exactly. However, we believe this study provides invaluable insight to the surgeon in terms of guiding a patient through shared decision making for treatment of their intermediate grade MEC.

## Conclusions

Mucoepidermoid carcinoma is a relatively uncommon disease, and those with intermediate grade tumors represent an even smaller subset of this group. Thus, a single site can only generate a small number of cases over time. Nonetheless, there are no clear guidelines for management of the uninvolved cervical lymphatics in this group. Based on our experience in 35 patients, we would suggest that an observational approach could be considered an option, and that recurrence in the cervical lymphatics would be an infrequent event in patients with intermediate grade mucoepidermoid carcinoma.

## Author Contributions

**Jake Langlie**, conception, design, acquisition of data, analysis, draft, and approval of manuscript; **Nicholas DiStefano**, conception, acquisition of data, analysis, draft, and approval of manuscript; **Carmen Gomez‐Fernandez**, conception, revising, and approval of manuscript; **Jaylou Velez‐Torres**, conception, revising, and approval of manuscript; **Jason Leibowitz**, conception, revising, and approval of manuscript; **David Arnold**, conception, revising, and approval of manuscript; **Donald Weed**, conception, revising, and approval of manuscript; **Francisco J. Civantos**, conception, design, analysis, revising, and approval of manuscript.

## Disclosures

### Competing interests

The authors declare no conflict of interest.

### Funding source

None.
